# Anæsthesia

**Published:** 1870-05

**Authors:** John G. Angell

**Affiliations:** New Orleans, LA.


### ANÆSTHESIA.

BY JOHN G. ANGELL, D.D.S., NEW ORLEANS, LA.

Read before the Southern Dental Association.

The honor of discovering an agent by which surgical operations could be effectually performed without the patient enduring the agony and torture that had heretofore attended the surgeons knife is due to the untiring energy of Horace Wells. Then to him be all the glory for the greatest boon yet bestowed upon suffering humanity, and though he met with an untimely end in his exertions to benefit others, his noble deeds will be engraved upon the tablet of memory and his name will live ages after we have passed away.

In administering an anæsthetic it is of the greatest importance to know the extent and manner of its action. Much has been said upon this subject, but more with reference to the physiology of its action upon the system, than to the method of tracing its effects and avoiding danger.

In this thesis it is not contemplated entering into the manner in which these agents act upon the system, as much as it is to trace the extent to which the system may be under their influence without incurring serious results.

How is this to be done? Not by the pulse, the respiration, time or quantity inhaled, but by the nerves, and their influence over the organs which they ramify. There is one established fact, and that is, all general anæsthetics are dangerous, aye, very dangerous in incompetent or inexperienced hands.

It then behooves those operators who contemplate using these agents, to proceed with care, investigate the various theories, and select very cautiously that which he thinks correct and safe. Anæsthetics may be divided into general (that which influence the whole system by inhaling the vapor) and local (confining its effects to the part to be operated upon). With the first the brain is cut off from the part to be operated upon. With the latter, the part to be operated upon is cut off from the brain. The writer will confine himself to general anæsthetics.

To administer these agents scientifically, it is absolutely necessary to be familiar with the anatomy and physiology of the system generally, and that of the brain in particular, as this organ is the great nervous center communicating with all parts of the body, so that an impression made at one extremity of a nerve is instantly made cognizant at the other. The brain is divided into four grand divisions: the cerebrum governing the mental faculties; the cerebellum governing coordination of motion; the pons varolii governing instinctive sensation, and the medula oblongata.

From these four grand divisions arise nine pair of nerves which are named according to their functions and are numbered according to their exit from the cranium as follows:

CEREBRUM.

1st, Olfactory, arises in the fissure of sylvius, and is distributed to the schneiderien membrane. Nerve of smell.

2d, Optic, arises from the thalamus opticus and tubercula quadrigemini and is distributed to the retina. Nerve of sight.

3d, Motor oculi arises from the crus cerebri and is destributed to the muscles of the eye except the external rectus and superior oblique.

4th, Patheticus, arises from the valve of the brain and is distributed to the superior oblique.

PONS VAROLII.

5th, Trifacial, arises from the pons varolii and is distributed to the orbit, eyelids, conjunctive, forehead, teeth, masseter, buccal, pterygoid and temporal muscles.

6th, Motor externus, arises from corpus pyramidal, and is distributed to external rectus muscle.

7th, Facial and auditory, distributed to internal ear, and muscles of face and neck.

8th, Pneumogastric, glosso pharyngeal and spinal accessory, distributed to muscles of respiration, &c.

9th, Hypoglossal, distributed to the tongue.

Anæsthetics when administered through the lungs to a sufficient extent, paralyze the nerves, that is, impair their power of performing their natural functions, by their action upon the brain. Affecting 1st the cerebrum; 2d, the cerebellum; 3d, pons varolii; 4th, the medulla oblongata, and thus the nine pair of nerves in the order in which they are numbered. Consequently, in administering an anæsthetic, its influence may extend entirely over the first three divisions and partially over the fourth without any serious result, provided we do not trespass too far upon the fourth.

Now, the question arises, how are we to judge how far the brain is brought under the influence of the agent, and how can we trace its effects upon the nerves without risking the life of our patient. Certainly not by the pulse, for that may not vary a single beat to the minute; not by stertorous breathing, for in many cases that is wanting; neither in the quantity inhaled, for what would scarcely affect one patient would kill another; nor in the time the system is under its influence for operations, in my own experience have varied from 10 minutes to 9 hours, but it is the face we must rely upon as the only true index. Why the face? Because the first seven of the nine pair of nerves that originate in the brain have branches distributed to muscles of the face, and as has been remarked when an impression is made upon one end of a nerve, as its origin, it is instantly communicated to the other, its point of distribution.

The first thing to be observed by an operator when called upon to administer an anæsthetic, is the temperament, constitution, general health of his patient, the nature of the operation to be performed, and then select such an agent as will best suit the case, and, having selected it, to satisfy himself that it is pure. 2d, the confidence of his patient; the promise without mental reservation to do as directed. This accomplished, and the napkins, water and antidotes conveniently arranged, he is ready to commence operations, always taking the precaution that the patient is not in an upright position, and especially to require, that perfect silence be observed.

All general anæsthetics have a primary and secondary action. The first exhilarates, and the second depresses the system; the latter is the state we wish to arrive at, consequently we push over the exhilarating stage as rapidly as possible. Of the many methods of administering them, that which is the most convenient and least formidable in appearance is the best if it will accomplish the object desired, such as handkerchief or napkin folded into a cone and well saturated with the agent on the inner side. The patient when inhaling should have the mind unoccupied, if possible, and should breathe naturally through both the mouth and nose, inflating the lungs at each inspiration to their full capacity. If, at the commencement, the fauces should be irritated so as to cause coughing, the "inhaler" should at first be held some distance from the mouth, and approach it gradually as soon as it ceases to be unpleasant. The first nerve that will be anæsthised, of the nine pair, is the olfactory—diminishing, and finally depriving the patient of the sense of smell; this is indicated by the patient remarking, it is not strong enough, they can not smell it, to replenish the napkin. Under such circumstances pay no attention to what they say, but tell them to take a full inspiration, or breathe "hard." Let them understand you have full confidence in yourself, and know what you are about. When the sense of smell is deadened, you may know the cerebrum, as far as the fissure of sylvius is anæsthised, as the olfactory nerve originates at that point.

The 2d, 3d and 4th pair of nerves, and the cerebellum will be affected in turn, as will be observed by the dilation of the pupil, contraction of the iris, and loss of co-ordination of motion. At this stage, there is still general sensibility, and instinctive winking. To overcome these it is necessary to anæsthise the pons varolii and part of the medula oblongata to the origin of the 7th pair of nerves. Then an operation may be performed without pain on any part of the body. If, on presenting the finger to the eye, the patient winks naturally, the pons is only slightly affected; if the winking is slow, it is gradually coming under the influence of the agent. At this stage, the patient will act as if intoxicated. If the patient is spoken to it should be in an undertone, or whisper, as the chorda tympani of the 5th, and portio mollis of the 7th are much excited, and the hearing is much more acute than when they are in a normal state. If the eyelids refuse to twinkle in response to tingling of the eyelashes with the finger, the pons is overcome, and the patient is in a condition to have the operation performed.

If the anæsthetic is continued until there is a relaxation of the muscles of the face, and all expression is lost, the 7th pair, which arises in the medula oblongata has succumbed to its influence. This is as far as it will be safe to advance, as we will then be verging upon the origin of the pneumogastric or 8th pair, which is the nerve of respiration, if that is paralyzed the lungs will cease to act, and death will ensue.

In extracting teeth it is not necessary to go beyond the pons, the origin of the 5th pair, as they furnish the Dental nerves. This will be indicated by the relaxation of the muscles of the extremities, and the eyelids failing to respond to the tingling of the eyelashes with the finger. In prolonged operations, it is necessary to push the anæsthetic to the origin of the 7th pair, or frequently renew its application, as the patient exhibits signs of recovering from its effects. After the operation has been performed, the next object is to restore the patient to consciousness as soon as possible, for this purpose aqua ammonia may be inhaled through the nose, or acetic acid may be placed upon the tongue, when there is danger from the lungs failing to act. Artificial respiration, as the dernier resort, must be persistently kept up with the galvanic battery, by placing the negative pole on the ensiform cartilège of the 7th rib, and the positive pole on the phrenic nerve, at the back of the neck. When the chest is inflated "let up" the battery by removing one of the poles, and if expiration is not free assist it by pressure upon the chest. Let the battery act 12 or 15 *times to the minute by the watch.* After this has been continued for a short time see if the lungs will act alone. If the patient gasps once, wait a minute for another gasp and sprinkle water upon the face and chest. Always guard against the tongue falling backwards into the pharynx and closing the glottis as it is liable to do if the position of the body is favorable to it during the "deep stage" of anæsthesia. Where it is available, nitrous oxide gas has the preference over all other anæsthetics, as it takes less time, more agreeable to inhale, and does not produce emesis, langour and weakness that generally follows the administration of chloroform and ether. Patients should take anæsthetics upon an empty stomach and care should be exercised to prevent the blood entering it during the extraction of teeth. More care is required to administer an anæsthetic to a patient to perform an oral operation than on any other part of the body and as Dentists are called to perform operations under its influence more than any other profession, it is important they should be familiar with the subject and not be under the painful necessity of having a physician present to guard against danger.

It is to be hoped that the time is not far distant when all within the fold of our profession will feel the importance of these truths and make it evident to the medical profession that we are not only their equals so far as administering anæsthetics is concerned, but that if there is any difference we are the superiors.

July 28, 1869.

